# Description of a Preparation of Introsuscepted Cœcum, Presented to the Royal College of Surgeons

**Published:** 1822-04

**Authors:** A. C. Hutchison

**Affiliations:** Surgeon Extraordinary to H. R. H. the Duke of Clarence, &c.


### MUSEUM PATHOLOGICUM.

NO. I.

**ART. III.**—*Description of a Preparation of Introsuscepted Cœcum, presented to the Royal College of Surgeons.* By A. C. HUTCHISON, Esq. Surgeon Extraordinary to H. R. H. the Duke of Clarence, &c.

DEAR SIR,

THE preparation of diseased and introsuscepted cœcum, which we examined together at the College a short time ago, I beg you will be pleased to present to the Board of Curators, in my name, for the museum. The particular appearances the preparation exhibits, you are much better able to describe than I am. I have to notice, however, that the introsuscepted cœcum, before its removal, was found on the *left* side, near to the commencement of the sigmoid flexure of the colon; and the whole cavity of the introsuscepted gut was completely filled by a cauliflower-like excrescence, growing from the villous coat. The inclosed note from Dr. Dunn, of Woolwich, and under whose immediate care the patient had been for a considerable period, is in answer to inquiries made by me into the symptoms manifested during the progress of this extensive and extraordinary disease.

The patient's name was B——, 73 years of age; had been a temperate-living man; and had resided on the coast of Africa for a period of twelve years. He was a tall stout man; and, besides the introsusception, the liver had the appearance, in colour, of having been boiled, but of its natural size, and free from abscess and tubercles. The pancreas was enlarged slightly, of a scirrhous hardness, and adhering to the stomach and duodenum. The villous coat of the stomach, particularly towards the pylorus, showed marks of inflammation. All the other viscera in the abdomen and thorax were free from the slightest mark of disease. Dr. Dunn was present when the body was examined. I am, dear Sir, your very obedient servant, A. C. H.

21, *Leicester-square; 16th Nov.* 1821.

*To W. Cliff, Esq.*

---

*Woolwich; 13th Nov.* 1821.

MY DEAR SIR,

As far as I recollect, aided by the information gained from the family, Mr. B——'s symptoms were principally confined to acute pain and weakness of the loins, defective appetite, and bilious suffusion of the whole surface; and, at intervals, when any sudden change of weather took place, every uneasy sensation became much aggravated. During the last stage of his illness, the stomach rejected all medicine. Soda-water, alone or with brandy, was the drink he liked. The nausea and fainting, which were the precursors of his death, must have arisen, in some measure, from the introsuscepted state of the bowels, inducing inflammation on the inner coats of the stomach. It was only a week, or perhaps less, before his death, that medicine lost its influence on the intestinal canal; which somewhat tends to show that this singular inversion of parts could not have existed for any length of time. Most abundant evacuations were brought away by active medicines, and sensible relief as constantly followed.

However, such a case is a rare proof of the frame of an old man withstanding such a mass of disease. Had the heart or lungs been, in any shape, affected, his days must have closed at a much earlier period.

R. DUNN.

*A. C. H. Esq.*

---

ART. IV.—*Tumor in the Cerebellum of a Child.* By the same.

